# Drug-eluting bead trans-arterial chemoembolization combined with microwave ablation therapy vs. microwave ablation alone for early stage hepatocellular carcinoma: a preliminary investigation of clinical value

**DOI:** 10.1007/s00432-021-03760-x

**Published:** 2021-08-17

**Authors:** Juanfang Liu, Wenguang Zhang, Huibin Lu, Hongbin Li, Xueliang Zhou, Jing Li, Xinwei Han

**Affiliations:** Department of Interventional Radiology, The First Affiliated Hospital of Zhengzhou University, No. 1, East Jian She Road, Zhengzhou, 450052 Henan People’s Republic of China

**Keywords:** Early stage hepatocellular carcinoma, Drug-eluting bead trans-arterial chemoembolization, Microwave ablation, Arsenic trioxide

## Abstract

**Purpose:**

To assess the clinical value of drug-eluting bead trans-arterial chemoembolization (DEB-TACE) combined with microwave ablation (MWA) vs. MWA treatment alone for early stage hepatocellular carcinoma (HCC).

**Materials and methods:**

Consecutive data from 102 HCC patients at early stage who were referred to our hospital from December 2014 to May 2016 were retrospectively collected. Forty-seven patients underwent DEB-TACE combined with MWA treatment, whereas 55 patients underwent MWA alone. After 1 month of treatment, the tumour responses of the patients were assessed using the mRECIST criteria. Treatment-related complications and hepatic function were also analysed for the two groups. In addition, overall survival (OS) and progression-free survival (PFS) were calculated and compared.

**Results:**

Patients in the combined treatment group (DEB-TACE combined with MWA) presented a better objective response rate (ORR) and disease control rate (DCR) compared with those in the monotherapy group (MWA treatment). The median OS and PFS were longer in the combined treatment group compared with the monotherapy group. Multivariate Cox’s regression further illustrated that DEB-TACE + MWA vs. MWA was an independent protective factor for PFS and OS. No serious treatment-related complications were observed in any of the patients.

**Conclusion:**

Combined treatment with DEB-TACE appeared to have advantages in prolonging OS and PFS compared to MWA. Therefore, combined treatment was efficient and should be strongly recommended to early stage HCC patients.

## Introduction

Hepatocellular carcinoma (HCC) is the third most common cause of cancer-related mortality worldwide (Bray et al. 2018). Consistent with the latest clinical practice guidelines for HCC issued by the European Association for the Study of the Liver (EASL), for early stage HCC patients who are defined as a single tumour ≤ 5 cm or three nodules ≤ 3 cm (Llovet et al. 2003), liver transplantation, surgical resection, and ablation are the recommended treatments (Galle et al. 2018). Recently, a minimally invasive and effective therapy has been highly praised by HCC patients. As the first choice for nonsurgical treatment, ablation has become a significant alternative treatment for small or early stage HCC. The efficiency of MWA has been confirmed repeatedly in the treatment of patients with tumours ≤ 5 cm (Kamal et al. 2019).

At present, arsenic trioxide (ATO) has been suggested to be an effective chemotherapeutic agent used primarily for solid tumours, such as HCC, colorectal, lung, bladder, breast, and pancreatic cancer (Huang et al. 2019; Kong et al. 2020; Kritharis et al. 2013; Wang et al. 2018). Trans-arterial chemoembolization (TACE) or DEB-TACE loading with ATO has been widely used and is effective in the treatment of human primary HCC. Given the advantage of avoiding systemic toxicity, drug-eluting bead trans-arterial chemoembolization (DEB-TACE) has been developed and widely used in combination therapy for HCC.

Combination therapy of TACE combined with thermal ablation yielded better treatment efficacy than monotherapy of MWA or TACE treatment in HCC patients, whereas DEB-TACE combined with MWA for early stage HCC patients has not yet been investigated. Thus, we hypothesized that DEB-TACE and MWA combination therapy might offer promising outcomes for early stage HCC patients.

## Materials and methods

### Patients

**Fig. 1:**
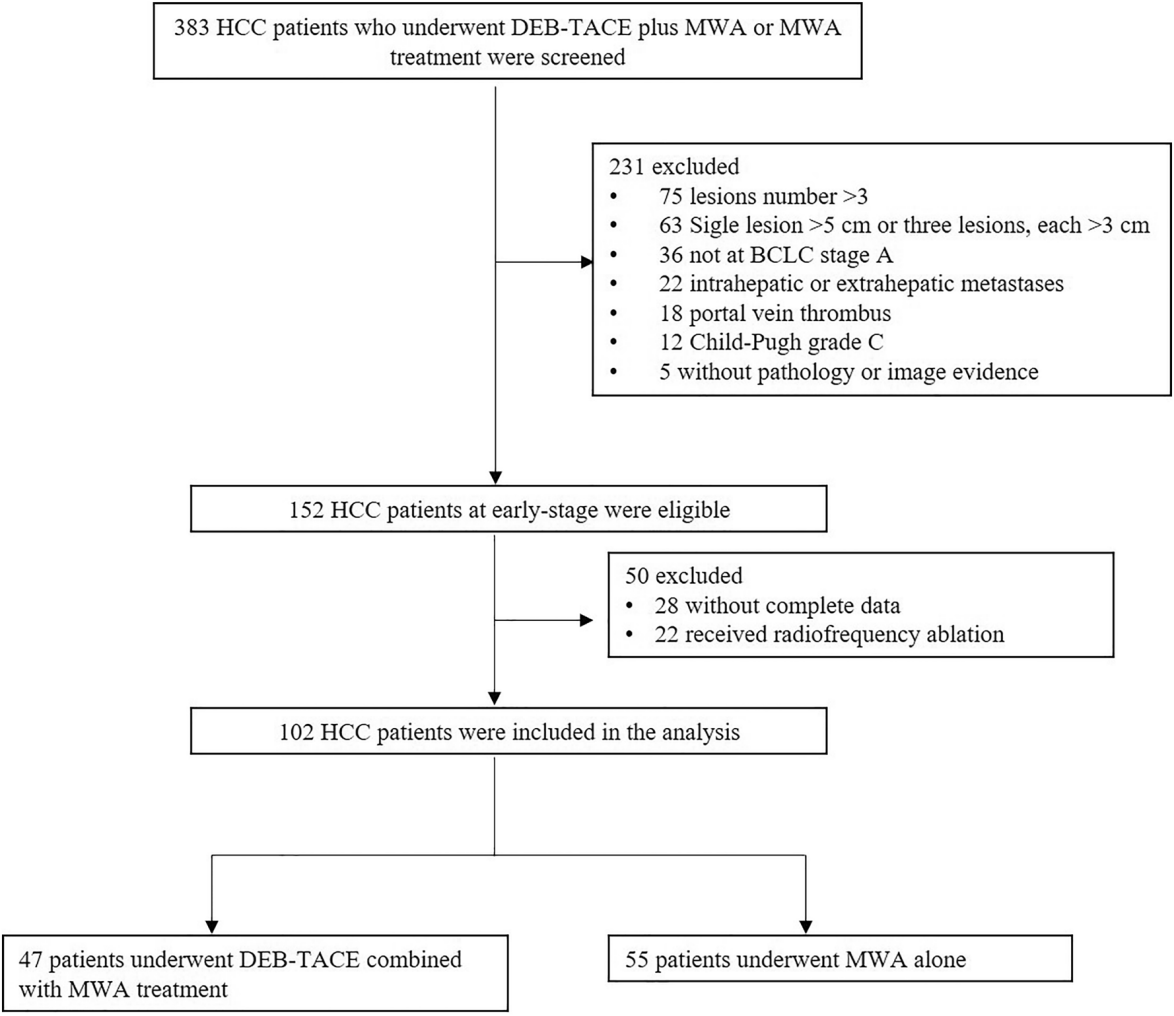
Study flow. *HCC* hepatocellular carcinoma, *DEB-TACE* drug-eluting bead trans-arterial chemoembolization, *MWA* microwave ablation

This retrospective study was approved by the institutional review board and was conducted in accordance with the Declaration of Helsinki. Written informed consent was waived, because the study was retrospective. From December 2014 to May 2016, a total of 383 HCC patients who underwent DEB-TACE plus MWA or MWA treatment were screened. 102 patients with small HCC were enrolled in our study. Forty-seven patients underwent DEB-TACE combined with MWA treatment, whereas 55 patients underwent MWA alone. The main inclusion criteria were (1) early stage HCC; (2) Child Pugh A; (3) Eastern Cooperative Oncology Group performance status (ECOG PS 0 or 1); and (4) completed data. The screening flow chart of inclusion and exclusion criteria were exhibited in Fig. 1.

### Treatment schedule

#### DEB-TACE treatment

Before DEB-TACE, the CalliSpheres Beads (Jiangsu Hengrui Medicine Co. Ltd., Jiangsu, China) with diameters of 300–500 μm were used as the carrier for loading with ATO (40–60 mg) (Beijing Shuanglu Pharmaceuticals, Inc., China). During the loading procedure, the mixture was shaken every 5 min at room temperature to enable the beads to fully load with ATO. Once loaded, iodine alcohol was added at a ratio of 1:1 into the above mixture for further use. The DEB-TACE procedure was performed as described by Wu et al. (2018) and Liu et al. (2019). The patients in the experimental group underwent DEB-TACE at least once, and treatment times should take into account tumour recurrence.

#### MWA treatment

MWA was generally performed by the same team of doctors 1 week after the DEB-TACE procedure. The patients were under conscious sedation and local anaesthesia. After anaesthesia was attained, the MWA probe (ECO-100AI10, ECO Microwave System Co, Nanjing, China) was inserted into the lesion under the guidance of CT with an output power of 50–60 W for 5–10 min. The ablation time should consider the depth, size and surrounding structure of the target lesions. Coagulation of the needle tract was performed to prevent bleeding or tumour seeding after ablation. Finally, a CT scan was performed after ablation to verify the extent of the ablation region as well as whether abnormal bleeding occurred.

#### Assessment of response and follow-up protocol

The primary endpoints included overall survival (OS) and progression-free survival (PFS). The time of OS was the period from the first interventional treatment to death or last follow-up. PFS was deemed the duration from the time of first interventional treatment to the time of disease progression or death or last follow-up. The secondary endpoint was tumour response, including the objective response rate (ORR) and disease control rate (DCR) as described in the modified Response Evaluation Criteria in Solid Tumours (mRECIST). ORR was defined as the percentage of patients who achieved CR or PR, and DCR was defined as the proportion of patients who achieved CR, PR or SD. Complications were observed clinically during admission and assessed by telephone interview after discharge. Complications were graded based on the Common Terminology Criteria for Adverse Events (CTCAE, version 3.0). Laboratory tests and abdominal ultrasound or enhanced CT/MRI were reviewed to assess tumour response at 7th day, first month, second month, third month and sixth month after the first treatment. Then, patients were followed up every 6 months. DEB-TACE + MWA or MWA was completed once more if tumour recurrence occurred. The last follow-up time was May, 15, 2021.

### Statistical analysis

Statistical analysis was performed using SPSS 22.0 statistical software (SPSS Inc., USA), and figures were created using GraphPad Prism 6.01 software (GraphPad Software Inc., USA). Categorical variables were expressed as numbers or percentages (%), and continuous variables were expressed as the mean ± standard deviation (SD) or median (25th–75th percentiles) as appropriate. Comparisons between two groups were determined by the Chi-square test, *t* test or Wilcoxon rank sum test. Survival analysis was performed using the Kaplan–Meier method, and differences in survival profiles between two groups were determined by the log-rank test. Multivariate Cox’s regression analyses were used to predict prognostic factors of PFS and OS. *P* value < 0.05 was considered significant.

## Results

### Patient characteristics

A total of 102 HCC patients at early stage were included in the present study. There were no significant differences in age, sex, Child–Pugh classification, cause of cirrhosis, tumour size, AFP level or tumour location between the two groups. The average tumour size was not different between the two groups, with 3.49 ± 0.88 cm in the DEB-TACE + MWA group and 3.48 ± 0.86 cm in the control (MWA only) group. The ratios of patients with 1, 2–3 nodules were 63.8%, 36.2% in the DEB-TACE + MWA group vs. 69.1%, 30.9% in the MWA alone group, and no significant differences were observed between the two groups.

### Tumour response

Tumour response was assessed on the basis of follow-up CT/MRI at the first month (M1) and sixth month (M6) after treatment. In the first month after treatment, the objective response rate (ORR) and disease control rate (DCR) were both slightly higher in the DEB-TACE + MWA group compared to the MWA alone group (100% vs. 94.5%, *P* = 0.106 and 100% vs. 98.2%, *P* = 0.355, respectively), and no significant differences were observed. However, at M6 after treatment, a significant difference in DCR (93.6% vs. 78.2%, *P* = 0.028) was noted between the two groups. Similarly, a more promising ORR (97.91%) was observed in the DEB-TACE + MWA group, which was significantly different from that (87.3%) in the control group (*P* < 0.05) (Table 1).

**Table 1:** Treatment response between the two groups

Response	CR	PR	SD	PD	ORR	P1	DCR	P2
M1								
Experiment group (*n* = 47)	45	2	0	0	100	0.106	100	0.355
Control group (*n* = 55)	50	2	2	1	94.5		98.2	
M6								
Experiment group (*n* = 47)	42	2	2	1	93.6	**0.028**	97.9	**0.047**
Control group (*n* = 55)	42	1	5	7	78.2		87.3	

*M1* first month after TACE therapy, *M6* sixth month after TACE therapy, *CR* complete response, *PR* partial response, *SD* stable disease; PD: progressive disease; *objective response rate (ORR)* CR + PR, *disease control rate (DCR)* CR + PR + SD

### Survival

**Fig. 2:**
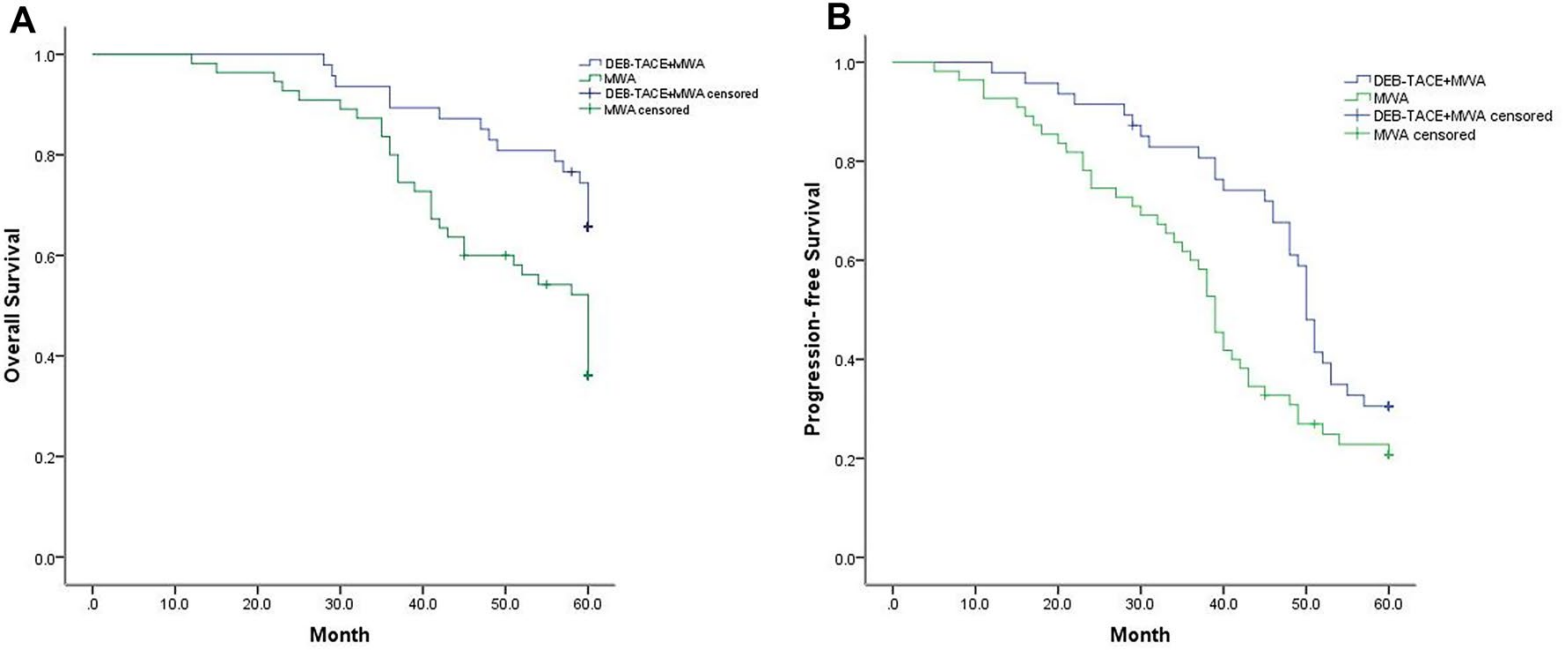
OS and PFS between DEB-TACE + MWA and MWAE groups. Kaplan–Meier method was used to assess OS and PFS. **A** DEB-TACE + MWA significantly prolonged the mean OS compared with MWA monotherapy. **B** Kaplan–Meier survival curve demonstrating longer mean PFS in the DEB-TACE + MWA group compared with the MWA alone group. DEB-TACE, drug-eluting bead trans-arterial chemoembolization; TACE, trans-arterial chemoembolization; MWA, microwave ablation; OS, overall survival; PFS, progression-free survival

The Kaplan–Meier method was used to assess the OS and PFS of HCC patients at early stage, and the difference between the DEB-TACE + MWA and MWA groups was determined using the log-rank test. At the end of the follow-up, a total of 50 patients died (16 patients in DEB-TACE + MWA group, 34 in MWA group). 32 patients occurred progression including 22 (51.1%) patients of intrahepatic recurrences and 8 (17.0%) patients of extrahepatic metastasis in DEB-TACE + MWA group. In the MWA group, there were 44 patients progressed including 33 (60.0%) of intrahepatic recurrences and 11 (20.0%) patients of extrahepatic metastasis. The mean OS was significantly improved in the DEB-TACE + MWA group compared to the MWA group (55.67 months; 95% CI 52.92–58.38 vs. 49.13 months; 95% CI 45.45–52.81; *P* = 0.003). The median OS was 60.0 months (inter quartile range, IQR 58.0–60.0 months) in the TACE + MWA group and 55.0 months (IQR, 37.0−60.0 months) in the MWA group. Similarly, the mean PFS was also significantly prolonged in the DEB-TACE + MWA group compared to the MWA alone group (47.61 months; 95% CI 43.89–51.32 vs. 38.56 months; 95% CI 34.26–42.86; *P* = 0.018). Based on the Kaplan–Meier curve, the cumulative survival rates at 1, 3 and 5 years were 100%, 89.4% and 65.7%, respectively, in the DEB-TACE + MWA group and 98.2%, 80.0% and 36.1%, respectively, in the MWA group (Fig. 2).

### Factors affecting OS and PFS

Univariable Cox proportional hazard regression analysis indicated that Child–Pugh class (B vs. A) (both *P* < 0.001) and numbers of lesions (2–3 vs. 1) (both *P* < 0.005) were associated with shorter OS and PFS. However, DEB-TACE + MWA vs. MWA showed a longer OS and PFS (*P* = 0.005 and *P* = 0.021, respectively) (Table 2). Multivariable Cox regression revealed that numbers of lesions (2–3 vs. 1) (both *P *< 0.001) independently predicted worse OS and PFS in HCC patients at early stage. While, DEB-TACE + MWA vs. MWA predicted longer OS and PFS (both *P* < 0.001) (Table 3).

**Table 2:** Univariate Cox’s proportional hazards regression model analysis for OS and PFS

Parameters	OS				PFS			
	HR	95% CI		*P*	HR	95% CI		*P*
		Lower	Higher			Lower	Higher	
Univariate Cox’s regression								
Age (≥ 60 vs. < 60)	1.427	0.800	2.544	0.228	0.866	0.531	1.478	0.642
Gender (Male vs. Female)	1.382	0.649	2.944	0.401	1.296	0.682	2.462	0.428
AFP (> 400 vs. ≤ 400 ng/mL)	1.322	0.759	2.303	0.324	0.901	0.570	1.424	0.655
Child–Pugh grade (B vs. A)	4.835	2.725	8.580	** < 0.001**	3.339	2.046	5.448	** < 0.001**
Number (2–3 lesions vs. 1)	5.431	3.061	9.637	** < 0.001**	4.844	3.013	7.790	** < 0.001**
Location (left vs. right)	0.970	0.455	2.066	0.937	1.130	0.621	2.055	0.690
DEB-TACE + MWA vs. MWA	0.423	0.233	0.767	**0.005**	0.581	0.366	0.921	**0.021**

*OS* overall survival, *HR* hazard ratio, *CI* confidence interval, *PFS* progression free survival

**Table 3:** Multivariate Cox’s proportional hazards regression model analysis for OS and PFS

Parameters	OS						PFS					
	*B*	Wald	HR	95% CI		*P*	*B*	Wald	HR	95% CI		*P*
				Lower	Higher					Lower	Higher	
Child–Pugh grade (B vs. A)	0.083	0.011	1.086	0.342	3.447	0.888	0.061	0.025	1.062	0.442	2.549	0.894
DEB-TACE + MWA vs. MWA	− 1.829	17.392	0.160	0.077	0.332	** < 0.001**	− 1.189	18.497	0.304	0.178	0.517	** < 0.001**
Number (2–3 lesions vs. 1)	1.898	19.415	6.667	3.052	14.564	** < 0.001**	1.765	27.283	5.840	3.057	11.158	** < 0.001**

### Adverse reactions and complications

The most common adverse events in patients who underwent DEB-TACE were gastrointestinal reaction, liver dysfunction, and bone-marrow suppression. Typical complications related to MWA included pain and fever. There was no significant difference in the incidence of complications related to MWA between the two groups (*P* > 0.05, all). Of the 47 patients who underwent DEB-TACE + MWA treatment, 12 patients had bone-marrow suppression, and 9 patients had nausea and vomiting. All adverse events and complications were classified as grade 1–2 according to Common Terminology Criteria for Adverse Event (CTCAE 3.0), including 9 patients who experienced pleural effusion (4 in the DEB-TACE + MWA group, 5 in the MWA group, *P* > 0.05), 8 patients who developed new ascites (3 in the TACE + MWA group, 5 in the MWA group, *P* > 0.05), and 10 patients who developed subcapsular liver haemorrhage (4 in the TACE + MWA group, 6 in the MWA group, *P* > 0.05). All patients were corrected after symptomatic treatment. No serious treatment-related deaths were observed (Table 4).

**Table 4:** Adverse reactions and complications

	DEB-TACE + MWA (*n* = 47)	MWA (*n* = 55)	*P*
Abdominal pain	19 (40.4)	16 (29.1)	0.229
Fever	15 (31.9)	16 (29.1)	0.757
Inappetence	14 (29.8)	11 (20.0)	0.252
Pleural effusion	4 (8.5)	5 (9.1)	0.918
Subcapsular liver haemorrhage	4 (8.5)	6 (10.9)	0.685
Liver abscess	3 (6.4)	3 (5.5)	0.843
New ascites	3 (6.4)	5 (9.1)	0.612
Liver dysfunction	13 (27.7)	11 (20.0)	0.363
Hypoalbuminemia	7 (14.9)	6 (10.9)	0.548
Bilirubin elevation	8 (17.0)	5 (9.1)	0.231
Nausea and vomiting	9 (19.1)	1 (1.8)	**0.003**
Bone-marrow suppression	12 (25.5)	0	** < 0.001**

## Discussion

TACE can selectively deliver chemotherapeutics to targeted lesions, leading to tissue necrosis by reducing the local blood supply of tumour lesions. However, the tumour recurrence rate was relatively high after TACE. Recently, many studies have revealed that patients with HCC treated with DEB-TACE tend to present a better response and tolerance than those who undergo TACE treatment (Liu et al. 2019; Woo and Heo 2015; Wu et al. 2018; Xiao et al. 2019; Zhou et al. 2018). DEB-TACE has the ability to control the speed of antitumour drug release, which can provide prolonged and sustained drug delivery and a high diffusion of chemotherapeutics into the local target lesion, resulting in a mild systemic reaction due to a lower concentration of chemotherapeutics in serum. As a chemotherapeutic agent, ATO is approved as a chemotherapy for HCC and other solid tumours in our country. Wang H demonstrated that locoregional therapy combined with ATO treatment could prolong the survival time of HCC patients (Wang et al. 2015). Similarly, our team previously demonstrated that DEB-TACE with ATO is more effective than TACE in patients with unresectable HCC (Duan et al. 2020).

Synergistic effect of TACE and MWA is ideal. Currently, combination therapy of TACE and MWA is effective and widely used in clinical practice (Li et al. 2020; Wang et al. 2019). In this study, we first assessed the efficacy and safety of DEB-TACE + MWA compared with MWA alone in early stage HCC patients. A study performed by Shi F et al. showed an ideal result: the OS rates were 100%, 79%, and 73% at 1, 3, and 5 years, respectively, in patients with HCC outside the Milan group who underwent TACE + MWA treatment (Shi et al. 2020). A retrospective study conducted by Zheng L showed better OS rates in the TACE + MWA group compared with the TACE alone group in patients with large HCC, with 1-, 2-, and 3-year OS rates of 85.9%, 59.8%, and 32.6% vs. 59.0%, 40.4%, and 11.4%, respectively (Zheng et al. 2018). Wei J and colleagues confirmed that TACE + MWA had superiority in prolonging median OS and reducing TTP compared with TACE + cryoablation (CRA) in treating unresectable HCC (Wei et al. 2020). In addition, our results showed that the OS rates at 1, 3, and 5 years in DCB-TACE + MWA group were 100%, 89.4% and 65.7%, respectively, which were similar with those reported by Li Z; the OS rates at 1, 3 and 5 years were 98.0%, 89.8%, and 74.3%, respectively, in patients with small HCCs treated with TACE + MWA (Li et al. 2020). Patients in the DEB-TACE + MWA treatment group achieved a prolonged mean OS (55.66 months) and an improved median PFS (47.61 months). In contrast, shorter median OS and PFS (49.13 months and 38.56 months, respectively) were obtained in the MWA alone group. At M6, more promising ORR (93.6%) and DCR (97.9%) were observed in the DEB-TACE + MWA group compared with the MWA group (78.2% and 87.3%, respectively). Recently, a meta-analysis suggested that TACE + MWA was efficient and safe for unresectable HCC patients with prolonged OS and tolerable adverse reactions (Wang et al. 2019). In this study, multivariable Cox regression analysis revealed that there was a significant correlation of shorter median OS and PFS with tumour numbers of 2–3, while DEB-TACE + MWA independently predicted a longer OS and PFS.

The results of this study are encouraging. TACE is the preferred treatment for HCC patients in all stages with well-tolerated liver function and is performed prevalently worldwide. However, TACE alone could not be sufficient for all HCC patients. Recently, MWA exhibits superiority in treating small HCC lesions (Li et al. 2021); nevertheless, this method is also affected by the cooling effect (Xu et al. 2019). The reasons are summarized as follows: (1) Tumour embolization caused by TACE could reduce the “cooling effect” of tumoral arterial blood flow. (2) The reduced or stabilized size of tumour after TACE would facilitate MWA treatment. (3) Ischemia and inflammatory edema, of tumour tissues occurred after TACE procedure, which had effect on increasing thermal effects of MWA. (4) MWA might destroy some hypovascular HCCs that were refractory to TACE procedures (Zheng et al. 2018). In this study, we substituted DEB-TACE for TACE given its better response and tolerance. Therefore, we administered DEB-TACE and MWA combination therapy, which can overcome these weaknesses and improve the efficacy of monotherapy.

Our study had several limitations. On the one hand, this was a retrospective study with a relatively small sample size, and the statistical power of our results might be mitigated. On the other hand, the breathing movement of patients might affect the accuracy of punctures under CT guidance. Therefore, further randomized controlled trials or prospective studies with larger sample sizes are needed to verify the results.

In conclusion, combination therapy of DEB-TACE with MWA prolonged the survival time of early stage HCC patients in comparison with MWA alone. Hence, treatment of DEB-TACE with ATO + MWA is safe and efficient, which may offer a promising new approach for the management of early stage HCC.

## Data Availability

The data used to support the findings of this study are available from the corresponding author upon request.
